# Buddha's ear illusion: Immediate and extensive earlobe deformation through visuotactile stimulation

**DOI:** 10.1177/20416695241262208

**Published:** 2024-07-23

**Authors:** Kenri Kodaka, Yutaro Sato

**Affiliations:** 12963 Nagoya City University, Japan

**Keywords:** nonproprioceptive ownership distortion, slime hand illusion, proprioceptive drift, illusory body-image deformation

## Abstract

The classical body ownership illusion, such as the rubber hand illusion, is achieved through appropriate proprioceptive displacement within temporal and spatial constraints that do not exceed the limits of proprioceptive flexibility. In the 2023 Best Illusion of the Year Contest, we introduced *Buddha's ear illusion* (BEI), which creates the illusion of owning a dramatically deformed earlobe through immediate visuotactile stimulation and seemingly challenges classical proprioceptive boundaries. The laboratory experiment examined the mechanics of this illusion, revealing a significant interaction between tactile earlobe pulling and visual miming that contributed to the enhanced perception of earlobe stretch. Importantly, 88% of the participants confirmed the illusory earlobe stretch (a rating of +4 or higher on a 7-point scale). More than half reported an earlobe descent of >10 cm within a 10-s visuotactile stimulation. The findings suggest that BEI operates on a distinct principle separate from proprioceptive modulation in contrast to classical ownership illusions.

The illusion of conventional bodily ownership, such as the rubber hand illusion (RHI; Botvinick & Cohen, 1998), has typically addressed the transformation of the body as a skeleton. A few studies report that subjective ratings related to the sense of ownership significantly diminish when the distance between the real and rubber hands is approximately 20–30 cm (Erro et al., 2020; Lloyd, 2007). In other words, the average proprioceptive error tolerance range in visual RHI is approximately 20–30 cm. This discussion suggests that further extending the conventional spatial boundaries of illusions is possible for the types of bodily transformation illusions that do not require proprioceptive displacement.

In 2022, we introduced the *slime hand illusion* (SHI) as a method for selectively deforming the skin region of the body image of the hand (Kodaka et al., 2022). SHI employs a layout of the mirror visual feedback (Ramachandran et al., 1995) and creates a sensation in which the skin is extended in a direction corresponding to the mirror image of the deforming slime. This perspective is achieved by pulling and stretching the skin of the hand behind the mirror, which is synchronized with the act of pinching and elongating the slime in front of the mirror. This illusion strongly evokes the sense of body-shape transformation, akin to studies reporting illusory head and nose extensions (Lackner, 1988), illusory changes in waist size (Ehrsson et al., 2005), and illusory finger extensions (Byrne & Preston, 2019). A distinctive feature of SHI compared to other illusions is its non-proprioceptive deformation; the measurement of the subjective sensations demonstrated that the SHI, at least subjectively, involves a “deformation of the skin without proprioceptive displacement.” Furthermore, in this experiment, the authors observed that the maximum displacement of subjective skin deformation during the experience averaged up to 29 cm in response to the visual deformation of the slime, which extended up to 40 cm. Considering that this illusion procedure occurs within an extremely short period compared with the average illusion onset in conventional RHI (approximately 15–20 s; Aimola Davies et al., 2013; Kalckert & Ehrsson, 2017; Lloyd, 2007), it strongly suggests that SHI emerges from a different principle than proprioceptive modulation, which was dominant in conventional RHI.

According to our hypothesis, the uniqueness of SHI is primarily attributed to the uncertainty in the spatial position information of the skin. Specifically, when the skin is pinched, the starting points that are being pinched can be identified on the homunculus map of the somatosensory cortex (S1; Penfield & Rasmussen, 1950). However, one cannot internally adequately perceive the extent to which the tip of the skin has elongated due to the limited number of cutaneous stretch receptors (Paré et al., 2003); thus, the distance remains elusive. The study infers that SHI operates by effectively leveraging this specific spatial cognitive limitation that is unique to the skin region. If this hypothesis is true, then SHI is likely to occur in other areas of the skin apart from the hand.

Motivated by these interests, the current study presented a new skin deformation illusion called Buddha's ear illusion (BEI) at the Best Illusion of the Year Contest 2023, where we applied SHI to the earlobe (Sato et al., 2023). BEI induces the sensation that the earlobe has stretched to a region where the other end of the rubber balloon (positioned between the earlobe and the fingers of an experimenter), is elongated in the same direction, while the experimenter simultaneously pinches and pulls the earlobe in a specific direction. BEI has two variations: one that uses a rubber balloon (top panel in Figure 1), and another in which it gives the illusion of pulling nothing in mid-air, as if stretching an elastic, transparent earlobe (bottom panel in Figure 1). The latter method corresponds to the report, as demonstrated in the aforementioned paper, that SHI can be induced even without the use of slime (invisible SHI), similar to the invisible hand illusion (Guterstam et al., 2013), the invisible finger stretching illusion (Byrne & Preston, 2019), and the long sixth finger illusion (Cadete & Longo, 2022). Based on observations of reactions of participants at the exhibition, the study infers that what happened to the skin of the hand with SHI is happening in the earlobe with BEI. However, at this point, no quantitative assessment of the illusion effect for BEI exists.

**Figure 1.**
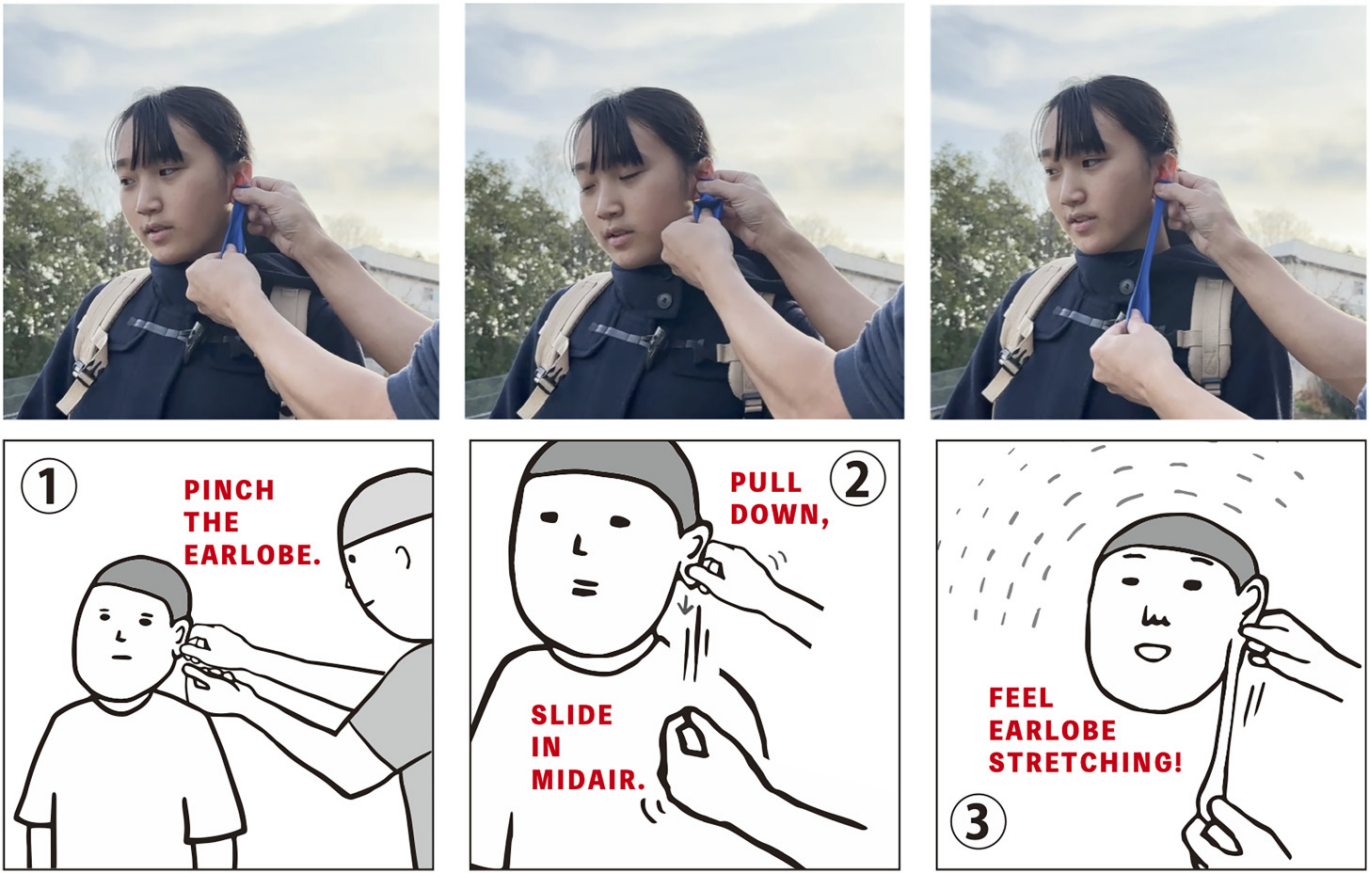
Top panel: typical process of inducing Buddha ear's illusion using a balloon; bottom panel: procedure of the invisible Buddha ear's illusion.

The main objective of this study is to examine the effects of visual and tactile operations in the invisible version of the BEI. Specifically, it assesses the effect of visual miming operations (performed by the lower hand of the experimenter) and tactile pinching and pulling operations (performed by the upper hand of the experimenter) in invisible BEI. For each sensory operation, contrasting conditions are designed (miming vs. staying for the visual operation, pulling vs. pinching for the tactile operation) for a total of four conditions for measuring the effects of BEI (refer to the top panel of Figure 2). To validate such effects, we conducted subjective evaluations through questionnaires (Experiment 1) and measured the spatial perception of the earlobe (Experiment 2). In Experiment 2, before and after the illusion, we measured the subjective positions of the upper and lower parts of the earlobe by having participants point to them with their eyes closed. This process was done to separate the deformation component of the skin portion of the ear from the overall earlobe movement. The hypothesis is that the strongest effect of BEI will occur under the condition of miming and pulling. The experiments provided strong support for the hypothesis and strongly substantiated that BEI is closely related to SHI.

**Figure 2.**
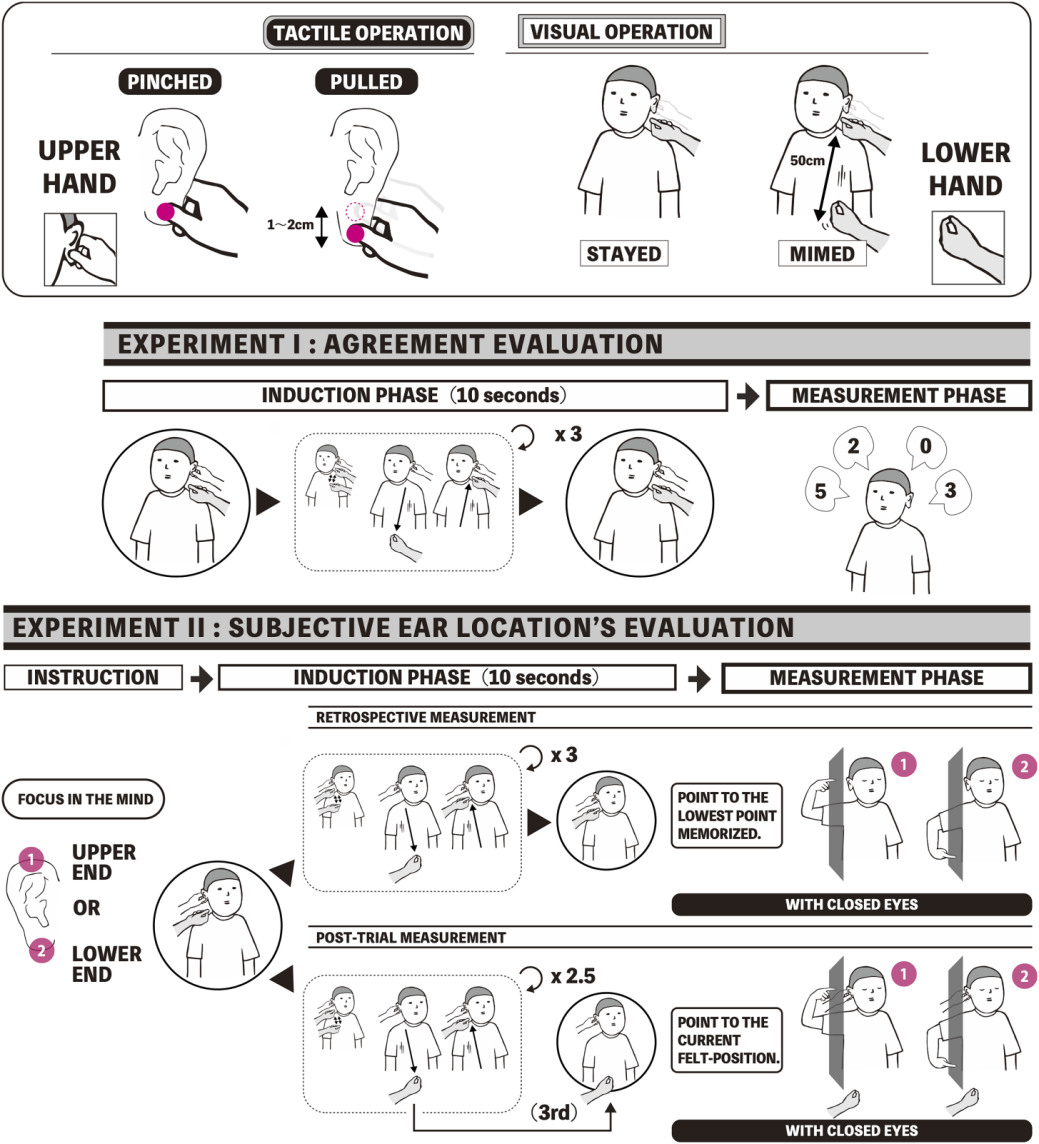
Top panel: the four visuo-tactile conditions, which consist of two tactile operations involving the upper hand of the experimenter and two visual operations associated with the lower hand of the experimenter. The simultaneous act of pulling the earlobe and moving the lower hand downward is performed during the Pulled × Mimed condition. Bottom panel: schematics of the experimental procedure for the two experiments with a focus on the specific condition in which the Mimed operation is applied. Notably, only the post-trial measurements were taken with the earlobe of the participants, and/or the lower hand of the experimenter is kept in a pulled-down position.

## Method

Sixteen students (1 female and 15 males, 18–29 years, *M* = 21.3) participated in the following two experiments. All participants received a book of tokens (1000 Yen) as compensation. The protocol was approved by the ethics committee of Nagoya-city University.

In this experimental setup, there are two types of tactile operations performed on the participant's earlobe, referred to as Pinched/Pulled, and two types of visual operations, referred to as Stayed/Mimed. Both manipulations are synchronously executed according to the experimenter's manual control using both hands. Specifically, the experimenter pinches or pulls the participant's earlobe using the upper hand (Pinched/Pulled), while simultaneously keeping the lower hand just below the earlobe or mimicking the motion of stretching the transparent earlobe using the lower hand (Stayed/Mimed). It is important to note that the illusion condition corresponds to the Pulled × Mimed condition.

As the initial state for all conditions, the experimenter's upper hand lightly pinched the earlobe of a participant, while the fingers of the experimenter's lower hand were positioned slightly apart from the participant's earlobe in a posture as if pinching the (nonexistent) transparent earlobe to keep it stationary. In each trial, the experimenter's upper hand lightly kept the participant's earlobe pinched (Pinched) or performed a series of actions that lasted approximately 2 or 3 s (Pulled) where the experimenter maintained the pinch on the participant's earlobe while gently pulling it downward by approximately 1 cm over approximately 2 s, then returning it to the initial state. The experimenter's lower hand, which was not in contact with the participant's earlobe, remained stationary below the participant's ear (Stayed), thus, maintaining the initial state, or it performed a series of pantomime-like actions (Mimed) where the experimenter's lower hand moved diagonally slightly forward and downward for a vertical distance of 50 cm over approximately 2 s from the initial state, and then quickly returned to its original position. In this Mimed operation, the experimenter's lower hand mimics the action of extending the participant's transparent earlobe further, which is similar to a pantomime.

The experiment compared a total of four conditions by combining two conditions related to the tactile operation with the experimenter's upper hand and two conditions related to the visual operation with the experimenter's lower hand. Before the tactile 1 cm and/or visual 50 cm roundtrip operation, which is referred to as “long strokes” and exhibited under the Pulled and/or Mimed conditions, a short initial movement that consists of two “short strokes” occurs. During this movement, the experimenter performs two consecutive instances of tactile stretching (by just less than 1 cm) and/or two consecutive instances of visual pantomime-like motion (by approximately 10–20 cm). This sequence of three consecutive strokes (two short strokes and one long stroke) is rapidly repeated three times. The total duration of these repetitions is approximately 10 s.

The participants stood in an upright position approximately several tens of centimeters away from a plain wall and began each trial in a relaxed posture while facing forward (toward the wall). The participants were explicitly instructed to maintain a forward-facing head position without any movement per trial and without closing their eyes. However, they were given the freedom to move their gaze as desired. As a result, the mimicked motion of stretching the transparent earlobe in the Mimed condition was consistently captured in the participant's peripheral vision.

### Experiment 1

Trials corresponding to each of the four conditions were conducted sequentially in a counterbalanced order among the participants on the left ear. Afterward, a questionnaire that consists of four items was promptly administered, containing the following statements.

1. I felt as if my earlobe stretched more than normal.
2. I felt as if I had an “invisible” skin in empty space.
3. I felt as if my whole ear was moved downwards.
4. I felt as if the entire area of my ear was shrinking.

Statements 1 and 2 were the illusion statements, while statements 3 and 4 originally served as controls for suggestibility and task compliance. The strength of each perceptual effect was rated using a 7-point scale (0 = not at all; 6 = very strongly agree). Note that Statement 3 was intended to assess the subjective sensation of proprioceptive drift across the entire ear. According to our hypothesis, the BEI mainly yields subjective earlobe deformation rather than movement of the entire ear, similar to the SHI. Therefore, we hypothesized that the significant effect of visuo-tactile synchrony, involving pinching and miming, would be observed only in statements 1 and 2. The entire procedure of Experiment 1 is depicted in Figure 2.

### Experiment 2

#### Baseline Measurement in Experiment 2

As a baseline measurement, the subjective height of the upper and lower ends of the ear in the pre-illusion stage is measured. Specifically, the experimenter touched the participants, whose eyes were closed, at the upper or lower end of the ear. The participants indicated the corresponding height on a measuring board using the right hand while keeping their eyes closed. These measurements were alternately conducted three times for the upper and lower ends of the ear, and the average of these measurements was taken as the baseline for the subjective height of the upper/lower end of the ear. The distance between the measurement board and the right ear of the participant is approximately 20 cm.

#### Retrospective Measurement in Experiment 2

Continuing in the same order as Experiment 1, the trials for the four conditions were sequentially conducted. Before each trial, the participants were instructed to pay attention to the subjective height sensation of the upper or lower end of the ear and to memorize the height at which it was most descended during the trial. They were prompted to close their eyes after each trial and report the memorized height by indicating it on the measurement board (Figure 2). One half of the groups first performed four trials with a focus on the upper end of the ear followed by four trials focusing on the lower end of the ear. The other half of the groups did the trials in the reverse order.

#### Post-Trial Measurement in Experiment 2

Subsequently, the participants were instructed to focus on the upper or lower end of the ear at the beginning of each trial; they then conducted the trials for each of the four conditions (a total of eight trials with four conditions, each with an upper- and lower-end focus). In the post-trial measurement, the ninth stroke involves a one-way movement only for the reciprocating movement of the Pulled and Mimed operations. Specifically, in the Pulled operation, the earlobe remains in the stretched position after pulling, while in the Mimed operation, the lower hand of the experimenter maintains its position after reaching 50 cm below without any additional movement. At the end of each trial, while maintaining the final state, the participants were promptly instructed to close their eyes. They were then asked to report the “current felt-position of the upper or lower end of their earlobe” by pointing to the appropriate positions on the measurement board. During the pointing process, the participants under the Pinched condition maintained the pinch on their earlobe, while those in the Pulled condition kept their earlobe pulled downward until the pointing was completed (Figure 2).

## Results

### Data Analysis

Before conducting the analysis, the subjective shift of the upper/lower end of the ear was calculated by subtracting the subjective location of the upper/lower end of the ear from the subjective baseline location of the upper/lower end of the ear for each condition. Then, the change in subjective ear size was calculated by subtracting the subjective shift of the upper end of the ear from that of the lower end of the ear for each condition. The analysis of the subjective shift of the upper/lower end of the ear was described in the Supplemental material.

We performed nonparametric analysis using two-way or three-way repeated-measures analysis of variance with an aligned rank transformation (ART ANOVA; Wobbrock et al., 2011). We also planned to conduct nonparametric pairwise comparisons using the ART-C procedure (Elkin et al., 2021) with Bonferroni's correction per condition. Statistical analysis was performed using R version 4.2.2 (R Core Team 2022). Significance was set at *p *< .05.

### Agreement Evaluation

**Figure 3.**
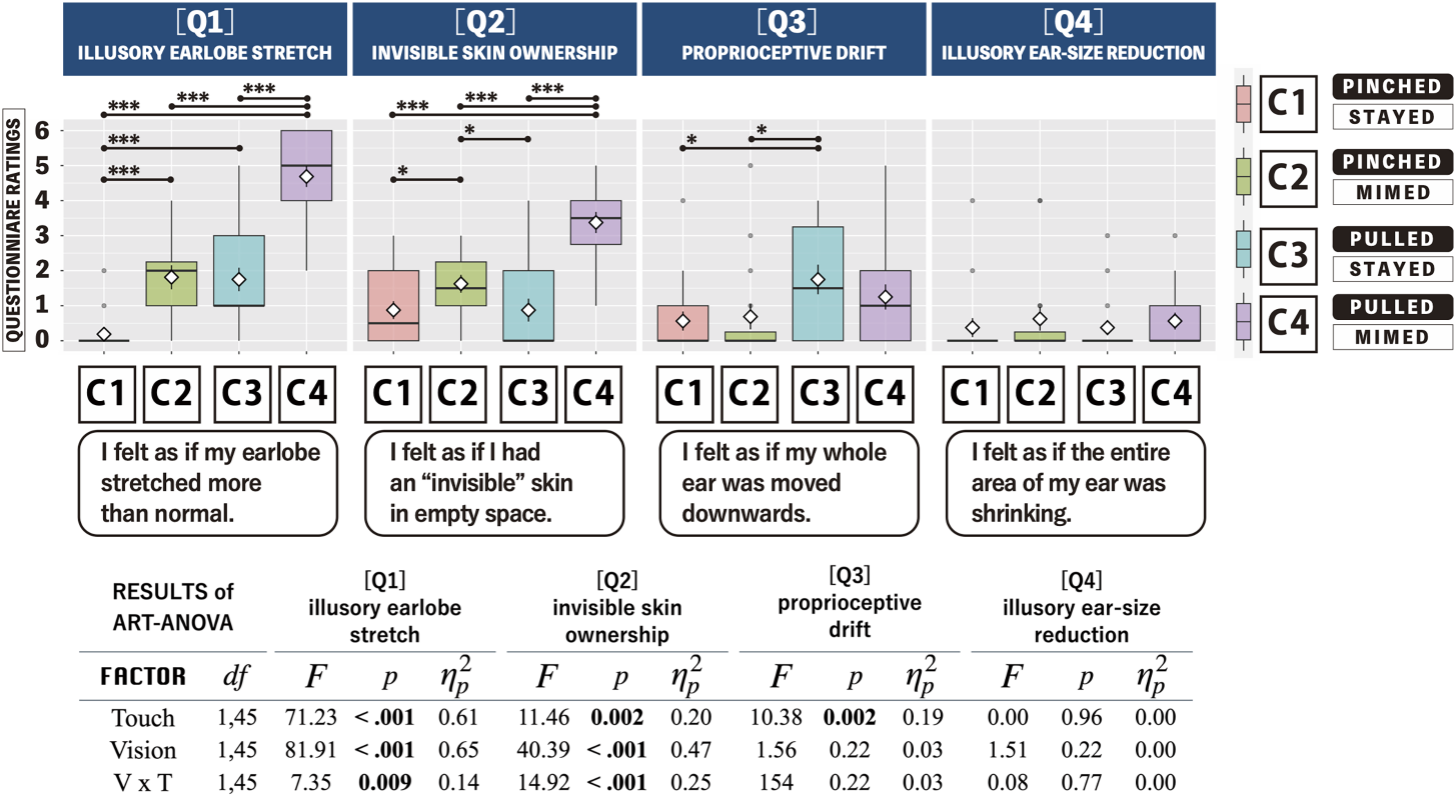
Average values of the subjective evaluations of the four questionnaire statements. Bold lines indicate median values, and white diamonds indicate the means. Error bars denote the standard error. The upper and lower limits of the box plots represent the 75th and 25th percentiles. Asterisks indicate significant differences between comparisons (*p < .05, **p < .01, ***p < .001). The table in the bottom panel displays the results of ART ANOVA.

In the two-way repeated-measures ART ANOVA (touch × vision), the study observed significant main effects of tactile and visual operations for two questionnaire statements (Q1: illusory earlobe stretch, Q2: invisible skin ownership). Furthermore, the interaction effect between tactile and visual operations was significant for these two statements. Regarding the statements for Q3 (proprioceptive drift) and Q4 (illusory ear-size reduction), a significant main effect was found only for the tactile operation in Q3, and no significant interaction effect was observed for Q3 and Q4. As follow-up analysis of the interaction effect, pairwise comparisons revealed that the ratings for the illusory skin stretch and invisible skin ownership in the Pulled × Mimed condition are significantly higher compared with the three other conditions (*p *< .001, for all combinations). Figure 3 illustrates the statistical details.

### Evaluation of Change in Subjective Ear Size

**Figure 4.**
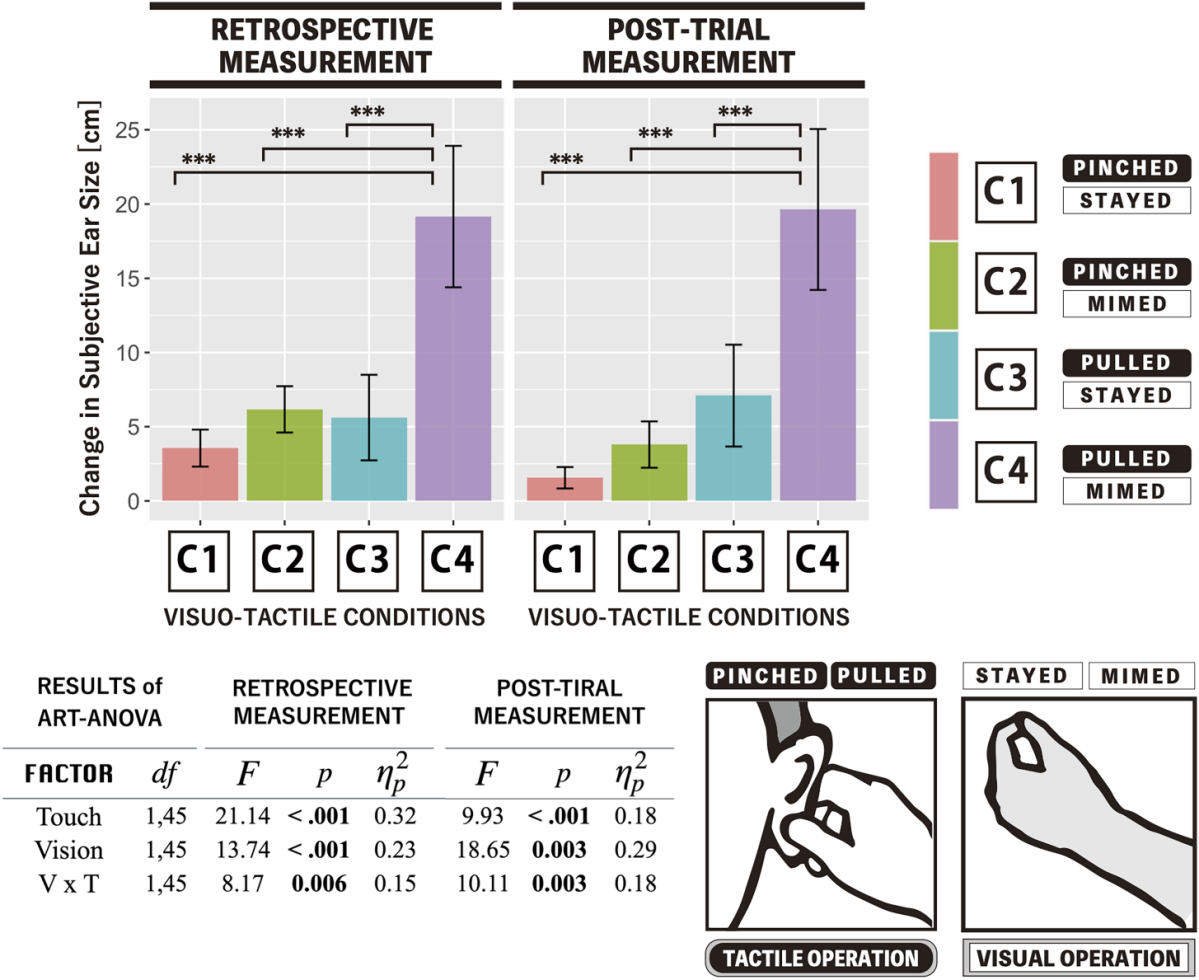
The average values of the change in the subjective ear size for two distinct measurements. Error bars denote the standard error. Asterisks indicate significant differences between pairwise comparisons (*p < .05, **p < .01, ***p < .001). The table in the bottom panel displays the results of ART ANOVA.

Two-way repeated-measures ART ANOVA (touch × vision) was conducted for the change in subjective ear size in each of the two types of measurements (retrospective and post-trial reports). The results revealed significant main effects for two factors, namely, tactile and visual operations, as well as a significant interaction between two factors, respectively, for each two measurements. To investigate the interaction effect, the study conducted follow-up pairwise comparisons among four conditions. In both measurements, the change in subjective ear size in the Pulled × Mimed condition was significantly larger compared to the other three conditions (*p *< .01 for all combinations), whereas there were no significant differences between any two conditions selected from among Pinched × Stayed, Pinched × Mimed, and Pulled × Stayed. Figure 4 provides the statistical details.

## Discussion

The results of the experiment provided statistical evidence that the tactile and visual procedures in BEI, in fact, enhance its illusionary effect in terms of subjective and behavioral indicators. A seemingly crucial aspect regarding visuotactile synchrony is that merely pinching the earlobe results in a highly restricted effectiveness of BEI. In fact, in the Pulled × Mimed condition, a substantial number of participants (14 out of 16) reported values of +4 or higher with regard to subjective ratings of “feeling that the earlobe stretched more than usual.” Alternatively, in the Pinched × Mimed condition, only two participants reported values of +4 or higher, while more than half reported values of +2 or higher, which indicates a moderately consistent effect. Furthermore, the change in the subjective ear size in the Pinched × Mimed condition is less than one-third when compared with that of the illusion conditions (6.16 vs. 19.16 in the retrospective measurement, 3.79 vs. 19.63 in the post-trial measurement). These results demonstrate that visuo-tactile synchrony with the vector of the force applied when pulling the earlobe is crucial for creating a strong sense of skin deformation in the context of BEI.

Notably, even in the illusion condition in which the earlobe is pulled, and visual pantomime is exhibited, only one participant reported a rating of +4 or higher for the sensation of the entire earlobe descending. Furthermore, when considering the retrospective and post-trial measurements, more than half of the participants reported that the lower end of their earlobes descended by more than 10 cm, whereas none described a descent of 10 cm or more for the upper end of the earlobe in any of the measurements (see the Supplementary material). These results are consistent with the notion that the subjective movement of the upper end of the ear corresponds to the movement of the entire ear, while the movement of the lower end of the ear corresponds to the deformation of the earlobe. In summary, BEI exerts a psychological effect that selectively deforms the earlobe region while keeping the center of the earlobe fixed in position through the synchronization of the traction vector applied to the earlobe with the visual miming of earlobe extension.

In typical circumstances, proprioceptive drift is measured by evaluating the difference between the period immediately before and after the induction of the illusion with the concealed rubber hand or with eyes closed. This measurement typically results in an average of a few centimeters in the majority of studies. In fact, among the 18 standard RHI experiments reported before 2012 (reviewed by Aimola Davies et al., 2013), no experiment reported that the average value of proprioceptive drift exceeded 6 cm. These results hold true even for experiments involving invisible hands or fingers; the average amounts of proprioceptive drift or deformation, as measured in these experiments, typically amounted to only a few centimeters at most (Byrne & Preston, 2019; Cadete & Longo, 2022; Guterstam et al., 2013). In the current study, the subjective location displacement, which was observed at the upper end of the ear under the illusion condition, was less than 5 cm, which falls within the range typically associated with traditional proprioceptive drift measurements. In contrast, the average of the subjective location displacement of the lower end of the ear, which was more than 20 cm, largely exceeded these classical measurements. In this post-trial measurement, the measurements were conducted with tactile stimulation applied to the target body parts. This methodology does not align precisely with conventional measurement techniques; thus, caution is necessary when making direct comparisons. Nevertheless, these results strongly suggest that the skin-based body image exhibits greater spatial plasticity than does the proprioceptive body image.

## Supplemental Material

sj-pdf-1-ipe-10.1177_20416695241262208 - Supplemental material for Buddha's ear illusion: Immediate and extensive earlobe deformation through visuotactile stimulation Supplemental material, sj-pdf-1-ipe-10.1177_20416695241262208 for Buddha's ear illusion: Immediate and extensive earlobe deformation through visuotactile stimulation by Kenri Kodaka and Yutaro Sato in i-Perception
