# Supplementary material for: Buddha's ear illusion: Immediate and extensive earlobe deformation through visuotactile stimulation
Source: Iperception. 2024 Jul 23;15(4):20416695241262208. doi: 10.1177/20416695241262208 (PMC11320686; doi:10.1177/20416695241262208)
Supplement: sj-pdf-1-ipe-10.1177_20416695241262208 - Supplemental material for Buddha's ear illusion: Immediate and extensive earlobe deformation through visuotactile stimulation [file sj-pdf-1-ipe-10.1177_20416695241262208.pdf]

Supplemental Data: Buddha's Ear Illusion: Immediate and Extensive Earlobe Deformation Through Visuotactile Stimulation

Kenri Kodaka and Yutaro Sato

Graduate School of Design and Architecture, Nagoya City University, Japan

Corresponding author

Kenri Kodaka

2-1-10, Kitachikusa, Chikusa-ku, Nagoya-shi, Aichi, 464-0083

kenrikodaka@gmail.com

+81-90-8386-7598

https://orcid.org/0000-0002-5426-6918

Results (Supplemental Analysis)

Evaluation of Change in Subjective Ear Location

Three-way repeated-measures ART ANOVA (ear position × touch × vision) was conducted for the change in subjective ear location in each of the two types of measurements (retrospective and post-trial reports). The results revealed significant main effects for three factors, namely, ear position and tactile and visual operations, as well as a significant interaction among all combinations of the three factors, respectively, for each two measurement. To investigate the interaction effect, the study conducted follow-up pairwise comparisons to focus on the impact of the ear position within each specific tactile and visual operation and the differences in the drift of the bottom of the ear among the four types of operations. In the retrospective report, the subjective shift of the bottom location of the ear was significantly larger than that of the top of the ears in two conditions, namely, Pulled × Stayed and Pulled × Mimed. However, this difference was nonsignificant in the Pinched × Stayed and Pinched × Mimed conditions. In the post-trial report, the four conditions yielded a significantly larger subjective shift at the bottom location of the ear compared with that of the top of the ear. In the retrospective and post-trial measurements, the subjective shift in the location of the bottom of the ear in the Pulled × Mimed condition was significantly larger than those of the three other conditions. However, the difference in the subjective drift of the top of the ear between the Pulled × Mimed condition and the three other conditions was nonsignificant. Figure 4 provides the statistical details.

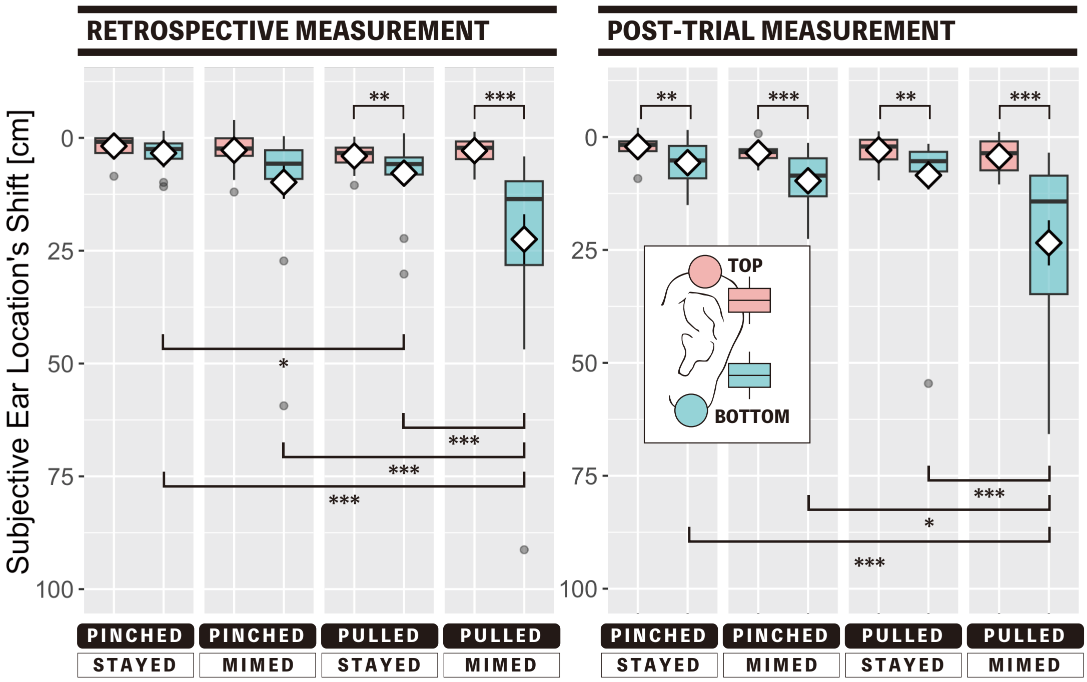

RESULTS of ART-ANOVA		PROSPECTIVE REPORT			POST-TRIAL REPORT		
FACTOR	*df*	*F*	*p*	η_p^2	*F*	*p*	η_p^2
Ear	1,105	71.91	< **.001**	0.41	107.61	< **.001**	0.51
Touch	1,105	46.00	< **.001**	0.30	26.83	< **.001**	0.20
Vision	1,105	29.81	< **.001**	0.22	56.74	< **.001**	0.35
E x T	1,105	23.96	< **.001**	0.19	16.19	< **.001**	0.13
E x V	1,105	34.75	< **.001**	0.25	34.41	< **.001**	0.25
T x V	1,105	11.91	< **.001**	0.10	19.36	< **.001**	0.16
E x T x V	1,105	21.34	< **.001**	0.17	20.10	< **.001**	0.16

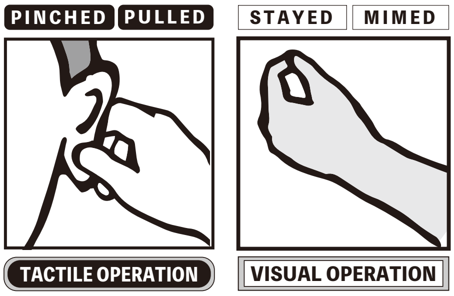

Figure 1.

The average values of the shift in the subjective ear location for two distinct measurements. Bold lines indicate median values, and white diamonds indicate the means. Error bars denote the standard error. The upper and lower limits of the box plots are the 75th and 25th percentiles. Asterisks indicate significant differences between pairwise comparisons (*$p < 0.05$, **$p < 0.01$, ***$p < 0.001$), where only the comparison between two ear positions under the identical operation and the comparison among the different operations for the ear's bottom is visible to avoid the visibility impairment. The table in the bottom panel displays the results of ART ANOVA.
